# Fiduciary-Free Frame Alignment for Robust Time-Lapse Drift Correction Estimation in Multi-Sample Cell Microscopy

**DOI:** 10.3390/jimaging10080181

**Published:** 2024-07-29

**Authors:** Stefan Baar, Masahiro Kuragano, Naoki Nishishita, Kiyotaka Tokuraku, Shinya Watanabe

**Affiliations:** 1 Graduate School of Engineering, Muroran Institute of Technology, Muroran 050-8585, Japan; sbaar@muroran-it.ac.jp (S.B.); gano@muroran-it.ac.jp (M.K.);; 2 Regenerative Medicine and Cell Therapy Laboratories, Kaneka Corporation, Kobe 650-0047, Japan

**Keywords:** image stabilization, optical flow, bright-field microscopy, fiduciary-free frame alignment

## Abstract

When analyzing microscopic time-lapse observations, frame alignment is an essential task to visually understand the morphological and translation dynamics of cells and tissue. While in traditional single-sample microscopy, the region of interest (RoI) is fixed, multi-sample microscopy often uses a single microscope that scans multiple samples over a long period of time by laterally relocating the sample stage. Hence, the relocation of the optics induces a statistical RoI offset and can introduce jitter as well as drift, which results in a misaligned RoI for each sample’s time-lapse observation (stage drift). We introduce a robust approach to automatically align all frames within a time-lapse observation and compensate for frame drift. In this study, we present a sub-pixel precise alignment approach based on recurrent all-pairs field transforms (RAFT); a deep network architecture for optical flow. We show that the RAFT model pre-trained on the Sintel dataset performed with near perfect precision for registration tasks on a set of ten contextually unrelated time-lapse observations containing 250 frames each. Our approach is robust for elastically undistorted and translation displaced (x,y) microscopic time-lapse observations and was tested on multiple samples with varying cell density, obtained using different devices. The approach only performed well for registration and not for tracking of the individual image components like cells and contaminants. We provide an open-source command-line application that corrects for stage drift and jitter.

## 1. Introduction

In this study, we present a fiduciary-free and robust image stabilization approach for simultaneous multi-sample time-lapse microscopy (SMSTM). Frame-to-frame image stabilization is an important task in many fields of microscopy such as fluorescence microscopy [1], single molecule localization, super-resolution microscopy [2], intravital video microscopy [3], etc. In particular, when estimating cell activity (cell movement, movement directionality, etc.), it is important to eliminate lateral jitter between neighboring frames of associate cells [4,5,6], to track them and compute their velocities. Here, the challenge lies in differentiating the individual cell’s motion from the motion of the field of view (FOV).

SMSTM is utilized in drug-related studies, to quickly evaluate multiple compounds distributed over several wells of a well plate, within the same time frame. During an experiment, the individual samples are scanned serially, which means that for each time step an image of each sample is taken and combined into a time-lapse sequence in post. The sample stage is moved laterally from well to well, to switch between a large number of cell-culture samples. Maintaining the same region of interest (RoI) for each sample over multiple switching cycles is restricted by long stage travel and the limited precision of the stage actuators. Relocating the RoI for each sample can be realized by utilizing fiduciary sample holders (also called image lock-plates) in combination with a internal device feed-back loop. This enables the microscopic system to reacquire the previous sample RoI and keep the image stable for the entire observation independent of directed or random cell movement. This method can be classified as an active stabilization method, where the stabilization is performed during observation. This has the advantage of locating the exact same location within the well plate, independently of the morphology or brightness of the observed objects (e.g., cells). A passive method is presented in this study. The frames are aligned in post, using computer vision to maintain the lateral RoI. In a more general sense, this task can be defined as image stabilization or fiduciary-free frame alignment for images without a clear point of reference.

Video stabilization and time-lapse observation can be achieved through image registration. This describes the process of identifying features in images or maps, with the goal of aligning them relative to a common coordinate system and origin. There are two types of image registration procedure: brightness-based and feature-based.

Brightness-based procedures are often used in applications containing unresolved objects, e.g., astronomical applications to match stars in the sky [7], or microscopic observations using fluorescence microscopy. Unresolved particles from fluorescence images were detected and utilized for frame-to-frame drift correction [8]. Stabilizing a set of images based on unresolved features has the advantage that, if features are point-like, the features can be localized very precisely, depending on the underlying broadening mechanism, which is either introduced by the media between instrument and the observed object or the limited resolution capabilities of the optical instrument. In either case, sub-pixel precise localization is possible using a point spread function [9].

Feature-based methods like phase-cross-correlation (PCC) and optical flow (OF) are where resolved features are used to determine the correspondence between a set of images. PCC is based on the Fourier shift theorem, in which the normalized cross-power spectrum is computed to factor out the phase difference created by two images that are shifted by the (u,v) to each other. The approach relies on frequency-domain representation and returns transversal displacement components (u,v), while most OF approaches, such as the Lucas–Kanade(LK) [10] and the TV-L1 method, rely on the fact that the flow (motion) is stable in a predefined, surrounding region of each pixel [11,12].

However, for applications using real-life data, previous studies claimed that it is necessary to separate moving from non-moving objects. This becomes especially difficult in datasets where many objects exhibit a directional group motion [2] that reassembles turbulent- instead of laminar flow. Chen et al. (2023) introduced a branch-and-bound algorithm to find subsets of point clouds as well as complementary information about cell shape and location to compute the matching likelihood of cell pairs in two imaging modalities [13]. This approach is based on feature detection and requires precise cell segmentation, performed using, e.g., Cellpose, which usually requires parameter tuning to achieve sufficient segmentation precision [14]. However, this approach is problematic because, at the time of writing, there is no single approach that can be considered robust enough to segment any image dataset and track all objects without requiring parameter tuning or retraining [15].

**Figure 1:**
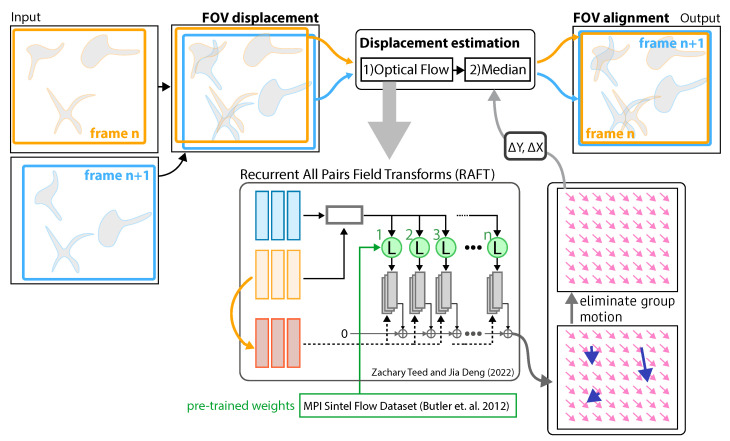
**Image stabilization:** An input containing a set of sequential images, where n is the frame number. The displacement information of the two frames is computed by first using recurrent all-pairs field transforms (RAFT) [16] trained on the Sintel dataset [17] to estimate the vector field describing the apparent motion (translation) of each pixel. The transversal displacement between frames (stabilized time-lapse observation) is determined by computing the median vector of the estimated vector field (pink arrows) and therefore eliminating the group motion of a set of sparse objects (blue arrows) within the RoI.

In this manuscript, we introduce a frame-to-frame matching approach for SMSTM, based on recurrent all pairs field transforms (RAFT) [16], as presented in Figure 1. Here, the translation vector between two images is estimated by first computing the displacement field and then computing the median of its x and y components. We were able to produce precise matching results for a range of time-lapse observations. The RAFT model trained on the Sintel [17] dataset performed better than traditional approaches such as PCC, LK, and TV-L1, but also significantly better than the RAFT model based on KITTI [18] without properly characterizing the individual cell movements. The sample stage of SMSTM exhibited only lateral (x-horizontal and y-vertical) movement. Therefore, transformations such as rotations, shearing, and non-linear holomorphic transformations were not considered. In the following, we will introduce the data apprehension approaches and elaborate on the methods for testing and comparing the different registration approaches. Next, we elaborate on the frame alignment workflow and how it is used to correct for frame drift. In the results section, we compare different image registration approaches on SMSTM and synthesized data with variable time spacing. Finally, we discuss the viability and elaborate on possible trade-offs.

A set of command-line applications to perform image stabilization, and to estimate and apply correction vectors, as well as the source-code and a sample of different datasets are publicly available at github http://www.github.com/stefanbaar/cell_align (accessed on 23 July 2024).

## 2. Materials

### 2.1. Cell Cultures and Reagents

We performed image stabilization for observations containing the following cell cultures and reagents.

#### 2.1.1. Cell Cultures

Human Astrocytes (HA, iPSC-derived, Normal, iX cells Technologies): 8–15 $\times \;10^{3}$ cells/well (medium: 150 µL), human iPSC-derived human astrocytes that display typical astrocytic morphology and express key markers of, e.g., GFAP, ALDH1L1 when cultured in Human Astrocyte Maintenance Medium (Cat# MD-0109-100ML).

#### 2.1.2. Reagents

QD-Aβ: 30 nM, Aβ: 5 µM, Plant extra (KNK XXX extra, MIT142 extra, KNK808 extra (final concentrations: 4 ng/µL)). DMSO (Control: final concentrations: 0.02%), Romaric Acid (RA) (Negative Control: final concentrations: 50 µM).

### 2.2. Simultaneous Multi-Sample Time-Lapse Observations (SMSTM)

**Figure 2:**
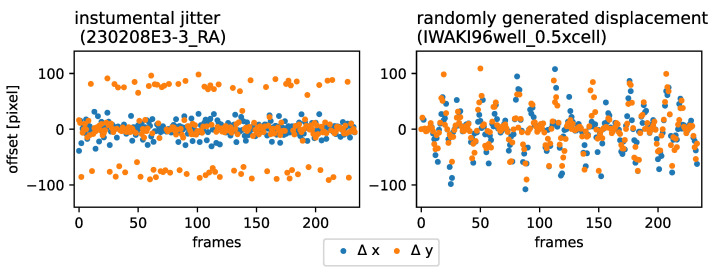
**Lateral RoI displacement synthesis:** (**Left**): time evolution sample of the instrumental jitter exhibited by Incucyte SX1. (**Right**): Randomly generated lateral jitter, introduced to unperturbed time-lapse observations obtained with an inverted Nikon Ti-E microscope.

Time-lapse imaging was conducted by the Regenerative Medicine and Cell Therapy Laboratories of the KANEKA CORPORATION, using an Incucyte SX1 (Sartorius Ltd., Goettingen, Germany) to perform SMSTM. This means multiple time-lapse observations were performed of samples. Recording was performed at every 20 min. Dynamics of QD-labeled A$\beta_{40}$ and A$\beta_{1-42}$ peptides were recorded with an Incucyte-SX1 (Sartorius, Bohemia, New York, NY, USA), and the exposure time was set to 90 h. A ×20 objective lens was used for image apprehension. The scan seating parameters were set as follows: Acquisition time: 400 ms, Iwaki-96 well plate (Catalog Numbers 3860-096) or image lock-late (Catalog Numbers 4379), three images per well. Estimated blob diameter: 5, threshold (RCU): 0.8. When a solvent (e.g., dimethyl sulfoxide, etc.) was necessary to prepare the dilution of plan extra, the same concentration of solvent was used for the pretreatment solution. The field of view (FOV) had a physical size of 0.88 mm × 0.65 mm and an image resolution of 1408 pixel × 1040 pixel. The frame rate was 20min/frame for human astrocyte observations and 60 min/frame for 2.5 d-neural cell observations. The individual frames exhibited a strong displacement, as presented in Figure 2, left. The lateral x-y displacement was caused by relocating the sample stage between each observation, to screen multiple samples. Focus drift (vertical displacement), as presented by Ma et al. (2023), was prevented by re-focusing before each observation [19].

### 2.3. Single-Sample Time-Lapse Observations

The data apprehension and data analysis software of the Incucyte SX1 is closed-source and therefore we were not able to produce a proper reference dataset utilizing the image lock-plate without applying the locking mechanism. Further, the fiduciary markers of the image lock-plate that the internal feedback mechanism of the Incucyte SX1 uses were not accessible. Therefore, we introduced an artificial frame-to-frame lateral jitter to serially apprehended datasets, which was physically unable to exhibit any frame jitter or drift. Next, the properties of the dataset are elaborated.

SH-SY5Y cells (0.1–0.2 × $10^{4}$ cells) were re-plated onto 0.1 mg/mL poly-D-lysine coated glass-bottomed 96-well micro-plates (IWAKI, Haibara, Japan). Cells were incubated overnight at 37 °C in humidified air containing 5% ${\mathrm{CO}}_{2}$. To inhibit actin polymerization and/or microtubule depolymerization, cells were treated with cytochalasin D and/or taxol at various concentrations. After incubation with inhibitors at 37 °C in humidified air containing 5% ${\mathrm{CO}}_{2}$ for one hour, cells were observed under, and time-lapse images were captured with, an inverted microscope (Ti-E; Nikon, Tokyo, Japan) equipped with a color CMOS camera (DS-Ri2; Nikon, Tokyo, Japan) and an objective lens (PlanApo λ 20×/0.75 NA; Nikon, Tokyo, Japan), resulting in a FOV with a physical size of 640 µm × 640 µm and an image resolution of 1608 pixel × 1608 pixel. During observation, cells were maintained in DMEM/F12 (1:1) (Gibco/ Life Technologies, Waltham, MA, USA) supplemented with 10% FBS and 100 µ/mL penicillin and 100 µg/M Lstreptomycin and warmed in a chamber set to 37 °C chamber (INUBTF-WSKM-B13I; Tokai Hit, Fujinomiya, Japan). Bright-field images were captured every minute for six to seven hours and exported using NIS-Elements AR software version 4.5 (Nikon). The images were captured in 8 bit RGB and exported by the camera (internally processed) in 8-bit greyscale.

### 2.4. Single-Sample Jitter and Stage Drift Synthesis

As a baseline and to evaluate our image stabilization approach and to compare it to previous methods, we introduced artificial translational frame jitter into single-sample time-lapse observations. As presented in Figure 2 Left, the simultaneously produced data taken with the Incucyte SX1 (without image lock-plate) exhibited a periodical jitter in horizontal (x) and vertical (y) directions with a primary amplitude $A_{p}$∼ 100 pixel and seemed to also exhibit an underlying modulation, exhibiting an secondary amplitude $A_{s}$∼ 20 pixel. We defined the translation transformation as shown in Equations (1) and (2) to imitate the lateral jitter-evolution of the sample stage, horizontally (x(t)) and vertically (y(t)).

(1) $$\left[\begin{array}{c} x^{\prime }\left(t+1\right)\\ y^{\prime }\left(t+1\right)\\ 1 \end{array}\right]=\left[\begin{array}{ccc} 1 & 0 & \delta x(t)\\ 0 & 1 & \delta y(t)\\ 0 & 0 & 1 \end{array}\right]\left[\begin{array}{c} x(t)\\ y(t)\\ 1 \end{array}\right]$$

with *t* being the frame number associated with a regular time interval and

(2) $$\left[\begin{array}{c} \delta x(t)\\ \delta y(t) \end{array}\right]=\vec{A}\left(t\right)\cos\omega_{1}t\cos\omega_{2}t\left[\begin{array}{c} x(t)\\ y(t) \end{array}\right]$$

with $\omega_{1}=0.5$ and $\omega_{2}=1.2$ and the displacement amplitude $\vec{A}\left(t\right)$ as a random vector $\left(\left[0,0\right]\leq \vec{A}\left(t\right)\leq \left[120,120\right]\right)\in R^{2}$. Using affine transformation with bi-linear interpolation, we produced a new set of displaced images that were used as the ground truth in this study. A comparison of the frame-to-frame displacement behavior between the Incucyte SX1 (without image lock-plate) observations and the synthesized data is presented in Figure 2 Right and Left, respectively.

## 3. Methods

### Displacement Estimation Using Optical Flow

**Figure 3:**
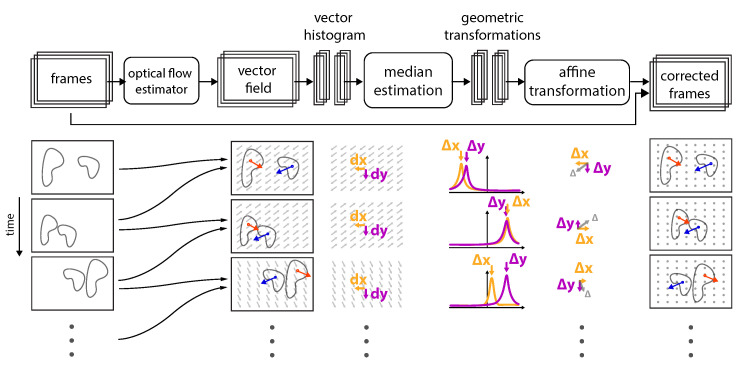
**Implementation overview:** Schematic of the data processing procedure used to correct frame-to-frame jitter is presented from left to right. For a set of frames, each frame and its following frame are compared and its dense vector filed is computed via optical flow. Notice that individual objects within the field of view (FOV) can move in random directions with random velocities (displacement amplitudes), independently of the underlying group motion (dx, dy). However, from the displacement vector histograms, one can identify a single peak that for each direction (Δx, Δy) characterized the displacement direction and amplitude, which is best characterized by the median. The median of the displacement field is then used to perform an affine transformation (in x and y) to correct each frame.

**Figure 4:**
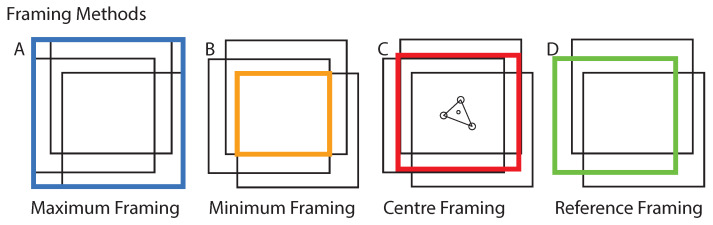
**Framing Methods:** An example set of three frames is presented. The four most plausible framing methods (**A**–**D**) are displayed. Maximum framing contains the information of all frames.

In this study, we utilized optical-flow to stabilize lateral frame-to-frame displacement. As visualized in Figure 3, each two neighboring frames were used to compute the vector field, also known as displacement (flow) maps, which are in principle the amplitudes ($A_{N,M}\in R^{N\times M\times 2}$) corresponding to the basis vectors $\vec{b_{y}},\vec{b_{x}}\in R^{2}$, where N and M are the pixel indices along $\vec{b_{y}}$ and $\vec{b_{x}}$. From the the set of histograms $H(\vec{b_{y}},\vec{b_{x}})$ corresponding to $A_{N,M}$, we computed the median of $H(\vec{b_{y}},\vec{b_{x}})$ to estimate time dependent displacement matrices Δ(t) with $\Delta_{y,x}\left(t\right)\in R^{2}$. The individual frames I(t,y,x) could be corrected $I^{\prime }\left(t,y,x\right)$ by applying the cumulative sum of the set of all displacement matrices, as follows $I^{\prime }\left(t,x,y\right)=I\left(t,x,y\right)\sum_{t}\Delta_{y,x}^{-1}\left(t\right)$. In addition, padding or cropping of each individual frame had to applied, depending on the reframing method displayed in Figure 4, showing examples for maximum, minimum, center, and reference framing.

Reference framing corresponds to the framing method where all frames were reframed according to the boundaries of a reference frame. Examples are presented in the Data Availability section of this study. Maximum framing was used to prevent information loss at the edges of each frame. Padding was also added to the raw frames to conserve the image FOV, for better comparability. The remaining area of each incomplete frame was filled with the median of the image brightness. For this study, we compared two optical flow algorithms (Lucas-Kanade [10] and RAFT [16]) to the established phase cross-correlation (PCC) approach, to determine the shift between two images.

## 4. Results

### 4.1. Simultaneous Multi-Sample Time-Lapse Observations

**Figure 5:**
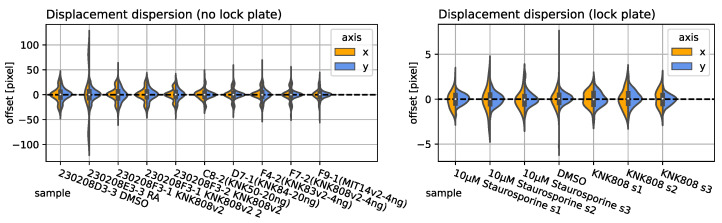
**Displacement dispersion:** Estimated with recurrent all-pairs field transforms and the median of the resulting vector field for ten sample observations without an image lock-plate (no position feedback) are presented on the (**Left**). Seven samples utilizing an image lock-plate and instrumental feedback loop are presented on the (**Right**). The horizontal and vertical displacement dispersions are colored in orange and blue, respectively.

In the following, we evaluate the abovementioned approaches on three different types of datasets for multi-sample observations with (no fiduciary markers) and without an image lock-plate (fiduciary markers), as well as single-sample observations with artificially introduced lateral misalignment in two directions (x and y). We evaluated the following nearest neighbor (NN) image drift and jitter for ten sample time-lapse observations with and without an image lock-plate and estimated the lateral NN offset using displacement maps obtained from RAFT (Sintel), as presented in Figure 5 right and left, respectively. The validity of the approach was visually confirmed by correcting each frame with the corresponding correction matrix.

Sample IDs present various inhibitors reducing cell activity (e.g., cell motility, protrusion density, projected area, etc.), as presented in previous studies [5]. However, the displacement amplitude and dispersion arise from instrumental influences and do not depend on any cell properties. As can be confirmed visually with the video data, the stabilization performed flawlessly for, e.g., observations with sparse (RA) and dense (KNK808v2) cell populations.

The corresponding sample videos are provided at github, accessed on 23 July 2024. Direct links and additional information for the example videos are presented in Table A1 of the Appendix A. For all samples, the video on the left presents the initial (displaced) time-lapse observation. The video on the right presents the stabilized time-lapse observation, where all frames were framed according to the maximum boundary. All samples exhibited no jitter or drift. The individual displacement evolution results for each sample are presented in Figure A2, Figure A3, Figure A4 and Figure A5 of the Appendix A.

The instrument (Incucyte-SX1) itself did not provide any reference data on the RoI offset without using an image lock-plate. Therefore, we took and corrected seven reference observations, utilizing an image lock-plate as reference. The manufacturer provided the offset error $\Delta_{ref}=\pm$5 nm, which corresponded to ±3.125 pixel. This was in agreement with the maximum displacement computed by RAFT, as can be seen in Figure 5 Right. We present the jitter, drift, and transversal correction amplitude evolution (from left to right) for two randomly chosen samples of RA and KNK808v2. Notice that the jitter and drift appear to be random and produced different patterns for both samples.

**Figure 6:**
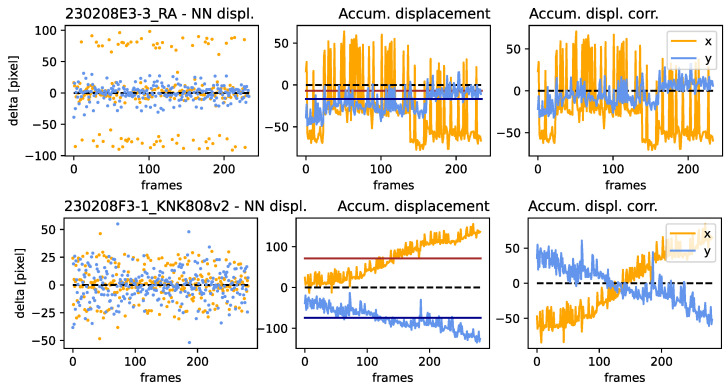
**Jitter, drift, and correction amplitude:** for two samples 230208E3-3_RA and 230208F3-1_KNK808v2 at the top and bottom, respectively. (**Left**): NN displacement amplitude per frame. (**Center**): The accumulated displacement, characterizing drift, where the red and black lines denote the median of the x and y components, respectively. (**Right**): The correction amplitude for each frame, determined by the accumulated displacement and its median. 230208E3-3_RA exhibits a relatively low drift in both directions (**upper center**), but a high frame-to-frame displacement amplitude in the x direction. 230208F3-1_KNK808v2 exhibits strong drift (**lower center**) and a lower frame-to-frame displacement amplitude for both directions.

The samples presented in Figure 6 were chosen because of their very different image drift characteristics and because they well represent the behavior of the remaining datasets, presented in Figure A2, Figure A3, Figure A4 and Figure A5. While the sample denoted RA exhibited strong frame-to-frame displacement in the horizontal direction, it did not exhibit strong directional drift, as presented in the center plot. In contrast, the sample denoted KNK808v2 exhibited a weaker frame-to-frame displacement, but exhibited a strong directional drift in both directions.

### 4.2. Synthesized Single Sample Time-Lapse Observations

**Figure 7:**
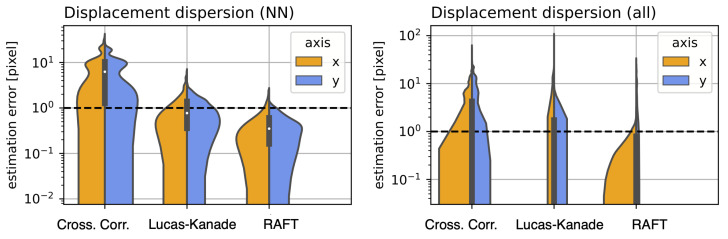
**Displacement dispersion:** for cross-correlation and the dense optical flow methods, based on Lucas–Kanade and RAFT. (**Left**): for the Nearest Neighbor (NN) displacement for each n and n + 1 frame. (**Right**): for all frame permutations of the entire observation.

Since there is no inherently precise method (as previously mentioned in Section 2.3) for evaluating the offset estimation accuracy for the Incucyte-SX1 system, we prepared a set of stable single sample time-lapse observations and displaced each frame randomly, as elaborated above. The results for the NN offset estimation error are presented in Figure 7. The individual NN displacement dispersion is presented on the right. On the left, we present the displacement dispersion for all frame distance permutations, not just the NN. This means we computed and compared the offset for each frame combination within the entire observation. The estimation error in both graphs was log scaled, due to the high variance when comparing the individual methods. Note that the order of magnitude between cross-correlation and Raft for the maximum estimation error was almost two.

**Figure 8:**
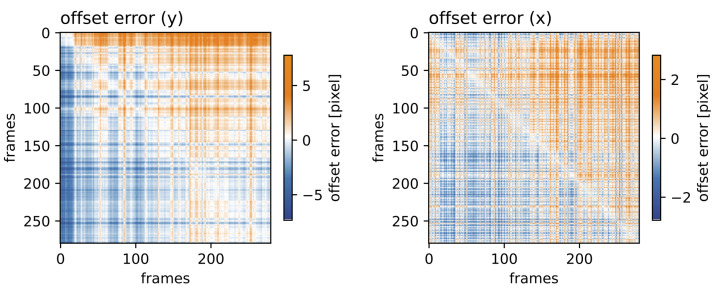
**Time distance-dependent offset error:** Distance matrix for all frame permutations of the vertical (**left**) and horizontal (**right**) components for the RAFT-based stabilization approach.

Next, we analyzed the coherence length of the RAFT-based stabilization approach. This was expected to strongly depend on the overall motility of the scene and incoherence (i.e., large alignment errors were expected). Figure 8 shows the offset error for all frame permutations for the vertical and horizontal components on the left and right, respectively. Even when aligning the two most distant frames (corresponding to a time difference of 250 min), the alignment error remained below six pixels for the vertical offset and below three pixels for the horizontal offset. The maximum distances are presented in the lower left corner and upper right corner of the matrix. The minimum distances are presented on the diagonal.

**Figure 9:**
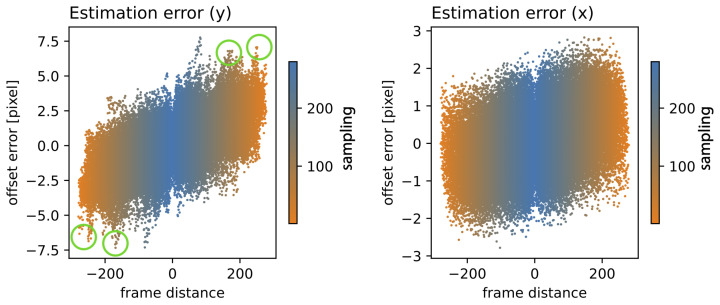
**Offset error in relation to the frame distance:** for various sampling ranges (orange to blue), for the vertical (**left**) and horizontal (**right**) components, utilizing RAFT stabilization. The positive vertical axis presents the correspondence between the nth and n+1st frame, the negative inverts the frame order and represents the n-1st and nth frame. The green circle indicate patterns that break point symmetry.

Figure 9 shows the relationship between the estimated (RAFT-based) offset error and the frame distance for the sampling ranges. The sampling range is indicated for low samplings in orange and high samplings in blue. The positive vertical axis presents the correspondence between the nth and n+1st frame, the negative inverts the frame order and represents the n-1st and nth frame. Note that the offset error is almost linearly related to the frame distance (which corresponds to the frame rate). Also note that certain features (circled in green) are not point-symmetric and different results were produced when changing the frame order. This means that RAFT was not strictly commutative.

## 5. Discussion

**Figure 10:**
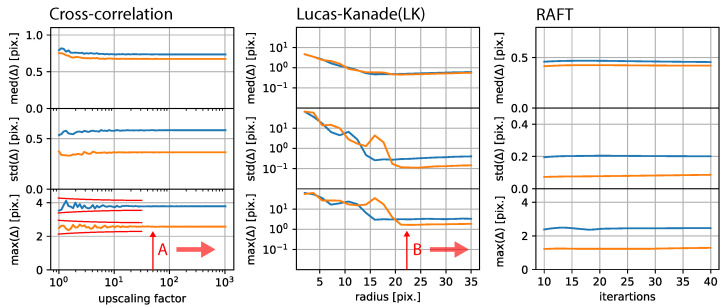
**Free parameter dependence:** (**Left**): Estimation error in relation to the upscaling factor of the phased cross-correlation-based stabilization approach. (**Center**): Estimation error for Lucas–Kanade-based stabilization, which mainly depends on the radius. (**Right**): RAFT-based stabilization error depending on the number of iterations.

In this study, we developed a method to correct 2D microscopic time-lapse observations below pixel precision, without the need for fiduciary methods, using RAFT. We compared our approach to established image registration and optical flow approaches. We visually confirmed the stabilization results, applied the stabilization to stable data, and tested our approach on synthesized time-lapse observations. Each registration approach had a set of variable parameters, and we estimated the best parameter from the distribution presented for each approach in Figure 10.

On the left-hand-side, cross-correlation-based stabilization exhibited strong fluctuations in terms of the maximum displacement error for low upscaling factors, which disappeared and become almost stable at an upscaling factor of 81. As presented in the center of Figure 10, Lucas–Kanade-based stabilization exhibited a minimum dispersion at a radius of 23 and was relatively stable for higher radii. RAFT-based stabilization only showed a very minor dependence of the displacement error on the number of iterations.

**Figure 11:**
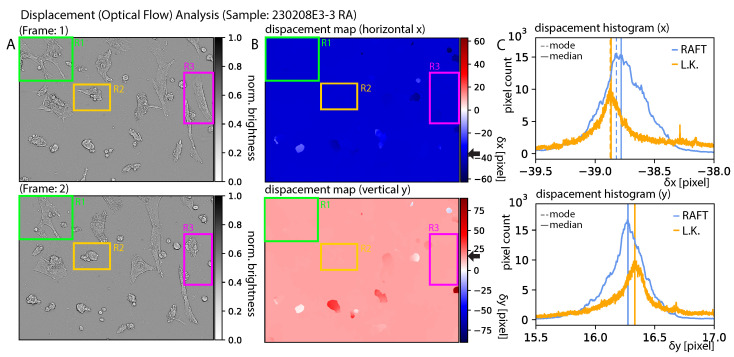
**Displacement analysis:** of sample 230208E3-3 RA. (**A**) shows the first (**top**) and second frames (**bottom**). (**B**) shows the corresponding displacement maps produced by RAFT. Regions R1 to R3 indicate cells that exhibited modality but were not registered in the (**B**). (**C**) Histograms corresponding to B for both RAFT (blue) and Lucas–Kanade (orange). The corresponding median and mode are indicated as solid and dashed lines, respectively. The black arrows near the color bars of (**B**) indicate the x-component of the median presented in the figures in (**C**).

Figure 11A presents the first and second frames of the sample denoted “230208E3-3 RA”. The displacement maps for the x and y directions are presented in B. Regions R1-3 indicate regions where cell movement was clearly visible but not recognized by RAFT. In general, the cell structure could be categorized as weakly modulated structures in comparison the image dimensions. The background features could be considered as strongly modulated features. RAFT seemed to be especially sensitive to small (high-frequency) features. The motility of individual cells was not reflected in the displacement maps. Therefore, the RAFT approach appeared to work well because it characterized the entire scene well, but not the individual cells. We conclude that the Sintel-trained model was not suitable for tracking individual cells. The movement of the individual cells could be categorized as elastic, which means that the cell morphology of the individual cells changed drastically from one frame to another. The detailed functions of the Sintel-based RAFT model were not fully comprehensible to the authors of this paper, which was caused by the optimization (training) of the many parameter model. The general process describing the optical flow method (e.g., Lucas–Kanade) was comprehensible, and it is generally known that optical flow is robust against shape-invariant translations, but not for structures exhibiting strong morphological changes, as presented in our study.

When analyzing the displacement histograms as presented in Figure 11C, it becomes inherently clear that this mode presented the best solution for an optimal dataset. However, in our approach, we chose the median (median = mode for symmetric distributions), since it better characterized flat tops, as presented in the top histogram of Figure 11C. For comparison, black arrows indicate the NN displacement (background of B) characterized by the median of the histograms (C).

**Figure 12:**
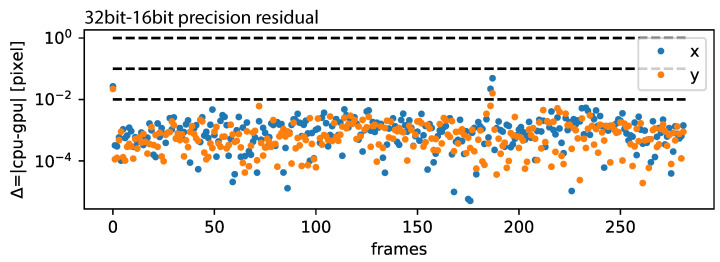
**CPU-GPU precision:** The residual of 32 bit/16 bit precision is plotted for each frame pare.

**Figure A1:**
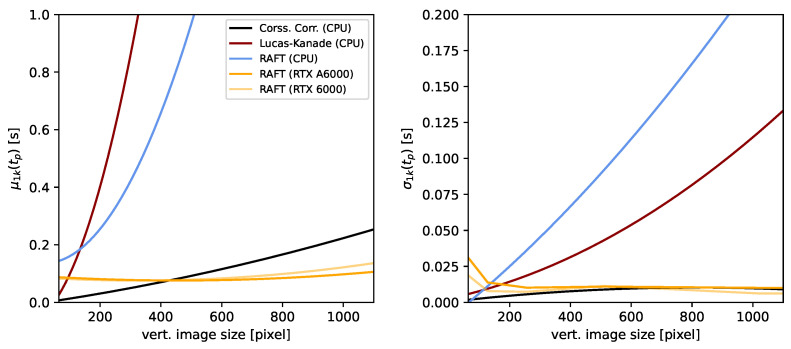
**Benchmark:** The average processing time for a set of image sizes and each image stabilization approach is presented on the left-hand side. The right-hand side presents the associated standard deviation for one thousand samples each.

**Figure A2:**
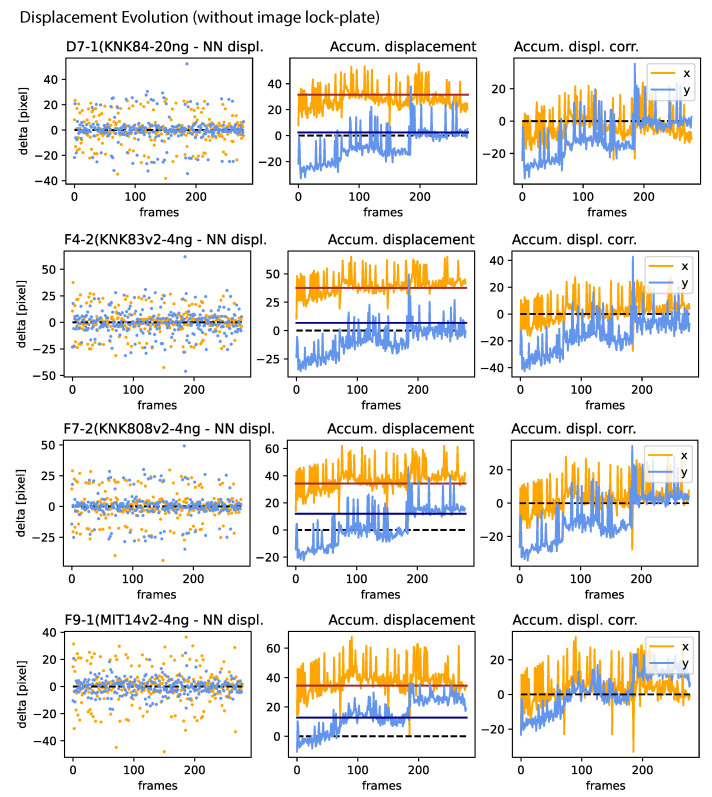
**Jitter, drift, and correction amplitude:** for all samples presented in Figure 5 (**Left**) for samples not utilizing an image lock-plate. (**Left**): NN displacement amplitude per frame. (**Center**): The accumulated displacement, characterizing drift, where the red and black lines denote the median of the x and y component, respectively. (**Right**): The correction amplitude for each frame, determined by the accumulated displacement and its median, in both directions.

**Figure A3:**
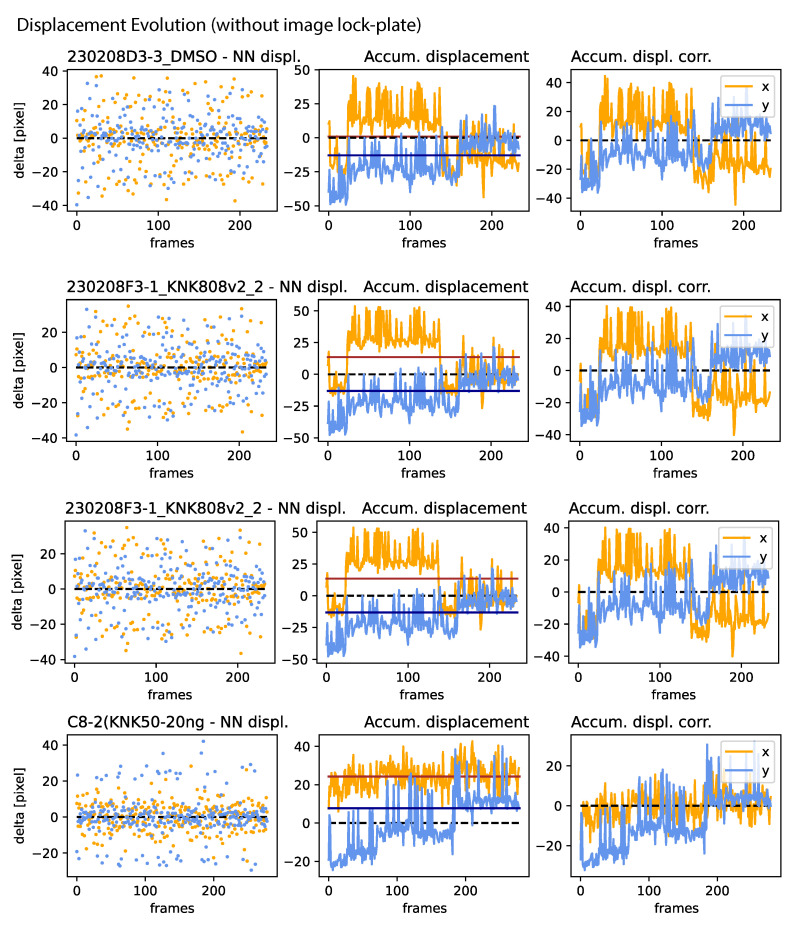
**Jitter, drift, and correction amplitude:** Continuation of Figure A2.

**Figure A4:**
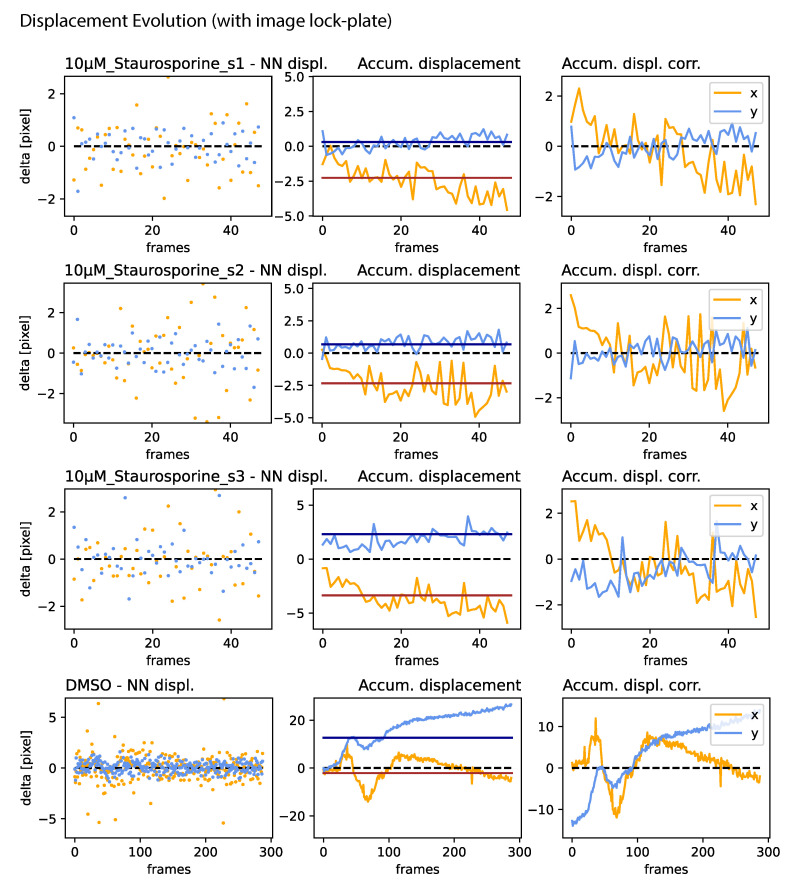
**Jitter, drift, and correction amplitude:** For all samples presented in Figure 5 (right for samples utilizing an image lock-plate. (**Left**): NN displacement amplitude per frame. (**Center**): The accumulated displacement, characterizing drift, where the red and black lines denote the median of the x and y components, respectively. (**Right**): The correction amplitude for each frame, determined by the accumulated displacement and its median, in both directions.

**Figure A5:**
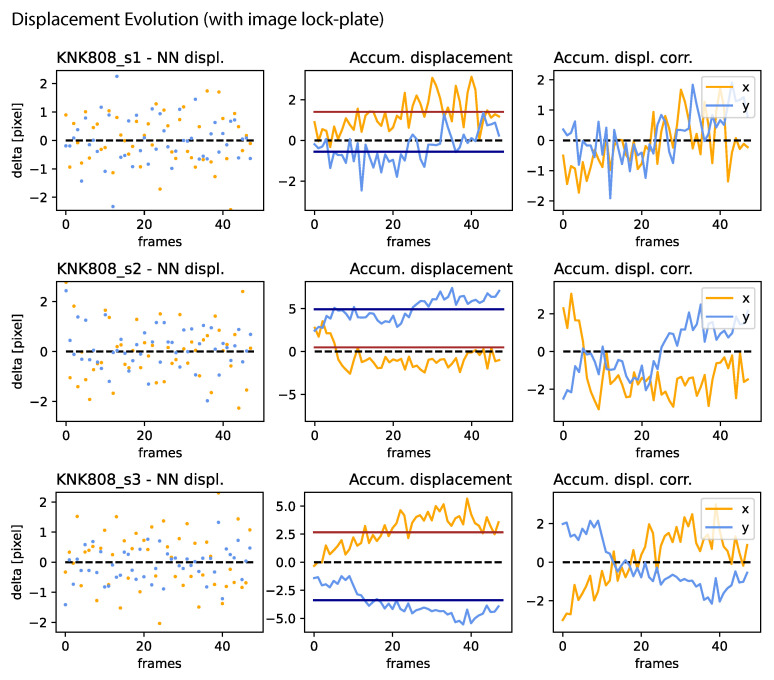
**Jitter, drift, and correction amplitude:** Continuation of Figure A4.

As presented in Figure A1 presented in the Appendix A, RAFT-based stabilization is computationally expensive and can be accelerated by using GPU-acceleration. This has the drawback that, for most commercial (affordable) GPUs, the floating-point precision (32 bit) is limited to half-precision (16 bit). We computed all samples presented above using half-precision. This did not affect the overall performance significantly, as presented in Figure 12, where the residual was mostly below 0.01 pixels and occasionally spiked to 0.1 pixels.

During the investigation stage of this study, we were not aware that the drift was only lateral and tried to solve for the holomorphic (off-axis) components of the transformation matrix (1). However, if off-axis components are truly zero, then a holomorphic solver will still produce minor off-axis values larger than zero and therefore not stabilize the frames correctly (because of the large degree of freedom). Therefore, our approach is limited to instruments exhibiting similar frame dislocation properties as the Incucyte-SX1 and cannot correct for holomorphic dislocations and transformations.

In general, image alignment strongly depends on the degree of feature change within images. In this case, this can be seen as the degree of motility and morphology of the individual cells within the FOV. We confirmed visually that the cells within the FOV are motile and undergo permanent morphological change. For future research, it will be necessary to compute cell motility and morphology precisely and investigate their relationship to stabilization accuracy. 

## Data Availability

Appendix A including the command-line application for time-series stabilization of sequential image and video data, the data analysis procedure in the form of jupyter notebooks, as well as a number of raw data samples are publicly available on github http://www.github.com/stefanbaar/cell_align (accessed on 23 July 2024).

## Appendix A

### Appendix A.1. Benchmark

The approaches presented in this study were benchmarked for various frame sizes, as shown in Figure A1. On the left, the average (μ) processing time for one thousand iterations ($t_{p}$) was computed for a range of frame sizes from 64 to 1200 pixel (vertical). The right side presents the standard deviation σ for each approach. The RAFT-based stabilization was computed using both CPU and GPU. The CPUs utilized in this study were 2×Intel(R) Xeon(R) CPU E5-2630 v4 @ 2.20 GHz. The GPUs used in this study were a single Nvidia RTX A6000 (24 GB) and Nvidia RTX A6000 (48 GB). All approaches were scaled exponentially with frame size. However, the RAFT-based approaches exhibited a nonlinear overhead for small frame sizes. The individual approaches were implemented using different libraries and programming styles and are not directly comparable in terms of performance. A more comprehensive performance evaluation in relation to the use case presented in this study needs to be carried out in future research.

### Appendix A.2. Video Data and Additional Information

The video data referenced in the results section (Figure 5) for the individual destabilized and stabilized datasets can be found, as previously mentioned, on github. Individual links are presented as follows, in Table A1.

Further, an extension of Figure 6, presenting the displacement evolution, for all remaining samples is presented in Figure A2, Figure A3, Figure A4 and Figure A5.

**Table A1:** **Supplementary information:** List of names and corresponding designations of initial and stabilized time-lapse observations. All links accessed on 23 July 2024.

Name	Supplementary Link	Image Lock
230208D3-3 DMSO	https://youtu.be/gazuq-znHJ4	✗
230208F3-1 KNK808	https://youtu.be/OyPupI3irXw	✗
230208F3-2 KNK808v2_2	https://youtu.be/IWGN8xM2RHk	✗
C8-2 (KNK50-20ng)	https://youtu.be/4qXPaIT1RyE	✗
D7-1 (KNK84-20ng)	https://youtu.be/pACRvUySuNw	✗
F4-2 (KNK83v2-4ng)	https://youtu.be/fNndG4kYBEY	✗
F7-2 (KNK808v2-4ng)	https://youtu.be/aDbReTa5uZA	✗
F9-1 (MIT14v2-4ng)	https://youtu.be/Q40rKkvLOqk	✗
DMSO	https://youtu.be/5anohk2LAzE	✓
KNK808_s1	https://youtu.be/qoY8zan9tLQ	✓
KNK808_s3	https://youtu.be/TP3ap7Ny21g	✓
KNK808_s2	https://youtu.be/f1UzS_9vm_s	✓
10 μM Staurosporine s1	https://youtu.be/qQgZPQsjmU8	✓
10 μM Staurosporine s2	https://youtu.be/yMiYH9i22lc	✓
10 μM Staurosporine s3	https://youtu.be/W0lkcbwbZhY	✓
